# Psychological distress in adolescents: prevalence and its relation to high-risk behaviors among secondary school students in Mbarara Municipality, Uganda

**DOI:** 10.1186/s40359-023-01039-z

**Published:** 2023-01-09

**Authors:** Michael U. Anyanwu

**Affiliations:** Department of Community Health, Mbarara University of Science and Technology, P.O Box 1410, Mbarara, Uganda

**Keywords:** Psychological distress, Risky behaviors, Adolescents, Substance use, Students, Uganda

## Abstract

**Background:**

Psychological distress among adolescents negatively affects their academic performance, relationships with family and friends, and ability to participate fully in the community. Stressful life events in low-income countries and risk-taking behavior of adolescents have raised concerns regarding the impact of psychological distress among adolescents. Therefore, the present study estimated the prevalence of psychological distress and examined the high-risk behaviors associated with psychological distress among Ugandan adolescents.

**Methods:**

A community based cross-sectional study was conducted among secondary school students in Mbarara Municipality, Uganda. Multistage cluster sampling was used to recruit 921 adolescents from 12 schools. Psychological distress was assessed using the 10-item Kessler Psychological Distress Scale (K10). Logistic regression was used with *p*-value estimating regression coefficient and 95% confidence interval for odds ratio and adjusted odds ratio (AOR).

**Results:**

The prevalence of psychological distress using the K10 was 57%. Logistic regression analyses showed that risky sexual behavior [AOR = 1.52; 95% confidence interval (CI) 1.02–2.28], substance use (AOR = 2.06; 95% CI 1.49–2.84) were associated with psychological distress. Students in mixed schools (comprising both sexes) (AOR = 1.94; 95% CI 1.19–3.15) and adolescents with chronic illness (AOR = 1.68; 95% CI 1.18–2.38) were more likely to report psychological distress.

**Conclusion:**

The prevalence of psychological distress among school-going adolescents is high. Risky sexual behavior, substance use as well as chronic illness were associated with psychological distress. In addition, the type of school was significantly associated with psychological distress. The findings suggest the need for policy makers and stakeholders in health and education sectors to institute measures that will address mental health issues among adolescents.

## Introduction

Mental health disorders are among the leading cause of ill-health and disability worldwide. According to World Health Organization (WHO), one in four individuals in the world are affected by mental disorder at some point in their lives and around 450 million individuals suffer from mental disorders worldwide [1]. Approximately 20% of the world’s children and adolescents have mental health problems [2] and mental health disorders account for 16% of the global burden of disease and injury among adolescents [3]. Psychological distress is a non-specific mental health condition that is characterized by anxiety, depression, and somatic symptoms. It involves feelings of vulnerability, sadness, fear, extensive worries, restlessness, negative thoughts and social isolation [4]. Mental health conditions (including psychological distress) are a public health challenge that affects daily activities of adolescents including their school and work performance, relationships with family and friends, and involvement in the community [2, 5]. Psychological distress has been associated with several negative consequences such as suicidal ideation and attempt [6, 7], poor academic performance [8] and poor physical health [9, 10] among adolescents.

Previous studies have indicated that psychological distress is common among adolescents. For example, prevalence rates of 35% in Canada [9] 54% [7] in Saudi Arabia and 40% in China [8]. In low-and middle-income countries, high prevalence of psychological distress has been reported among adolescents. Another cross-sectional study among students in India estimated 10.5% mild psychological distress, 5.4% moderate psychological distress, and 4.9% severe psychological distress [8]. Also, a study conducted in Zambia reported 15.7% of psychological distress among adolescents [11], while a study in Tanzania reported 20.6% single psychological distress and 10.3% multiple psychological distress [12]. In Uganda, a study conducted among refugee adolescents reported 12.3% psychological distress [7] while another Ugandan study reported 83.2% psychological distress among orphaned youths and youths experiencing sexual exploitation [13].

Adolescence is a period characterized by high-risk behaviors [14, 15]. High-risk behaviors such as substance use and risky sexual activities have been associated with psychological distress and other mental health problems among adolescents [9, 16, 17]. Previous studies have shown that alcohol use among adolescents is associated with psychological distress [18–20]. Substance use have also been associated with psychological distress among school-going adolescents in studies conducted in Norway, Zambia and Tanzania [11, 12, 21]. Moreover, previous studies have associated risky sexual behavior with psychological distress. Risky sexual behaviors such as early sexual debut, multiple sex partners and inconsistent condom use have been associated with psychological distress among adolescents [12, 17, 22–24].

Uganda is a low-income country where other health issues are prioritized over mental health due to limited resources [25, 26]. Moreover, stressful and undesirable life events continue to raise concerns regarding the magnitude of mental health problems in the country [27].

Previous studies in Uganda have focused on other mental health problems with limited studies conducted on psychological distress among students. In addition, prior studies examining adolescent mental health have concentrated on adolescents living with HIV/AIDS, as well as other vulnerable and marginalized population [16, 28–32]. Guided by the biopsychosocial (BPS) model of health [33], the present study estimated the prevalence of psychological distress and examined the high-risk behaviors associated with psychological distress among secondary school students in Mbarara Municipality, Uganda.

It is important to know the magnitude of psychological distress among students before the initiation and implementation of intervention strategies. The present study will provide baseline data on psychological distress among secondary school students, needed to institute prevention and control programs. In addition, the study findings can be helpful in understanding the implications of psychological distress among adolescents, and providing impetus for policy makers, stakeholders in health and education sectors to initiate policies that will address mental health issues among adolescents in Uganda.

## Methods

The data used in this study were part of a research project evaluating gambling disorder among secondary school students in Uganda. The study was a cross-sectional survey conducted in 2019 at Mbarara Municipality, a town located in the South-western part of Uganda and the main administrative and commercial center of Mbarara District.

### Sample

To achieve a representative sample of secondary school students in the Municipality, multistage cluster sampling was used. The first stage involved random selection of two schools from each of the Municipality’s six divisions with each division represented by one public and one private school. At the second stage, students were randomly selected from each class in the secondary school using the class register. A total of 921 students from 12 schools out of 35 registered schools in the Municipality were selected using a proportion of 10.2%, 95% confidence interval, 3% margin of error, and design effect of 1.826. The sample size was calculated by Kish Leslie formulae and the result multiplied by the design effect [34]. Secondary schools with both ordinary and advance classes were included in the study and students that had not been in school for at least 2 months or spent an academic session in the school were excluded in the study.

### Measures

#### Sociodemographic characteristics

The survey collected information on a number of sociodemographic characteristics: age, gender, religion, family background, class of student, school type, and caretaker/guardian. Participants also answered questions on owning a mobile phone (yes/no), and ever failed a subject in an exam (yes/no).

#### Psychological distress

Psychological distress was assessed using the 10-item Kessler Psychological Distress Scale (K10). Items are rated on a five point scale and evaluate the frequency of depressive and anxiety symptoms over the past 4 weeks. Psychological distress was categorized into normal, mild, moderate, and severe psychological distress. Scores range from 10 to 50, with score of 0–19 categorized as normal, 20–24 as mild distress, 25–29 as moderate distress and scores above 29 categorized as severe psychological distress [35, 36]. The K10 has been shown to be a reliable and valid tool for screening of adolescents in epidemiological surveys with Cronbach’s α coefficient of 0.86 [20], receiver operating characteristic (ROC) curve of 0.87–0.96 [35], and it has been used extensively in Sub-Saharan Africa [20, 37–39].

#### Substance use

This was assessed using the Alcohol, Smoking and Substance Involvement Screening Test (ASSIST), a tool validated by World Health Organization for assessing the use of alcohol, tobacco products and other psychoactive drugs. The tool was adapted, using only the second question which assessed the use of tobacco products, alcoholic beverages, cannabis, amphetamine type stimulants, inhalants, sedatives, hallucinogens, opioids and other substances in the past 3 months. A participant that has never used any of the substances was classified as “no substance use” and those that have used any of the substances were classified as “substance use” [40, 41].

#### Risky sexual behavior

The sexual behavior of the participants was assessed with questions regarding being sexually active, condom use, and ever being pregnant. The behavior was categorized as either: “no risky sexual behavior” or “risky sexual behavior”. Participants that answered “no” to being sexually active or “yes” to being sexually active but “no” to them or their sexual partner ever being pregnant and “yes” to consistent use of condoms were categorized as having “no risky sexual behavior”.

#### Chronic illness

The chronic illness or condition of the participants was assessed using the single question “Do you have a chronic (long lasting) illness or health condition?” with yes or no response.

#### Social support

Two questions were used to assess social support; “Are there family and friends that you can go to for advice and support?” and “Are there elders or community leaders that you can easily go to for advice and support?” Social support was classified into “no social support” and “Having social support”. Any “yes” answer to one of the questions was categorized as having social support.

### Data collection

Under the supervision of research assistants, students completed the self-administered survey in English language in a selected hall during the school period. Research assistants explained the objectives of the study to the students and assured them that all information was anonymous and that answers will not affect their grades in any way. Students that did not want to participate in the study were allowed to leave the hall where the survey was conducted. Written informed consent was obtained from the participating students and the legal guardian of students aged below 18 years before the survey was administered. The study was approved by the Research Ethics Committee of Mbarara University of Science and Technology with the IRB number of 07/03-19.

### Analysis

Data analysis was carried out using STATA Version 12.0. All participants that did not complete the K10 were excluded. Descriptive statistics was used to examine the participants’ characteristics. The prevalence of psychological distress was determined by the scores of the students on the (K10). Unadjusted and adjusted logistic regression analyses were carried out to identify the factors associated with psychological distress. All variables with a *p*-value of < 0.05 in the bivariate logistic regression analysis were included in the multivariable logistic regression model.

## Results

### Socio-demographic characteristics of study participants

A total of 921 students participated in the study. However, 15 students were excluded due to missing information on psychological distress (1.6%), leaving a final sample size of 906 participants. The minimum and maximum age of the participants was 11 years and 25 years respectively and the mean age was 16.9 (SD = 2.1). A higher proportion of the participants were male (53.6%), boarding students (75.9%), Catholics (36.7%), and attended mixed schools comprising of both sexes (86.7%). The socio-demographic characteristics of participants are shown in Table 1.

**Table 1:** Socio-demographic characteristics of participants

Variable	Normal	Mild distress	Moderate distress	Severe distress	Total
	n (column %)	n (column %)	n (column %)	n (column %)	N = 906 (column %)
	n = 390	n = 223	n = 163	n = 130	
*Age*					
Below 18 years	228 (64.4)	115 (56.7)	79 (53.4)	60 (54.0)	482 (59.1)
18 years and above	126 (35.6)	88 (43.3)	69 (46.6)	51 (46.0)	334 (40.9)
*Gender*					
Female	184 (47.3)	94 (42.5)	78 (48.2)	62 (48.1)	418 (46.4)
Male	205 (52.7)	127 (57.5)	84 (51.8)	67 (51.9)	483 (53.6)
*Type of student*					
Boarding	280 (73.5)	169 (77.2)	132 (83.5)	92 (71.3)	673 (75.9)
Day	101 (26.5)	50 (22.8)	26 (16.5)	37 (28.7)	214 (24.1)
*Type of school*					
Private	211 (54.1)	123 (55.2)	93 (57.1)	71 (54.6)	498 (55.0)
Public	179 (45.9)	100 (44.8)	70 (42.9)	59 (45.4)	408 (45.0)
Mixed (both sexes)	320 (82.0)	195 (87.4)	150 (92.0)	121 (93.0)	786 (86.7)
Single-sex	70 (18.0)	28 (12.6)	13 (8.0)	9 (7.0)	120 (13.3)
*Residence*					
Urban/town	220 (60.6)	111 (54.4)	88 (59.1)	59 (48.8)	478 (57.1)
Sub-urban	143 (39.4)	93 (45.6)	61 (40.9)	62 (51.2)	359 (42.9)
*Religion*					
Catholic	123 (31.9)	79 (35.4)	72 (44.4)	56 (43.4)	330 (36.7)
Moslem	38 (9.9)	25 (11.2)	15 (9.3)	6 (4.7)	84 (9.3)
Protestant	80 (20.8)	44 (19.7)	25 (15.4)	23 (17.8)	172 (19.1)
Pentecostal	89 (23.1)	45 (20.2)	34 (21.0)	32 (24.8)	200 (22.3)
Other	55 (14.3)	30 (13.5)	16 (9.9)	12 (9.3)	113 (12.6)
*Family type*					
Both parent alive	326 (84.9)	179 (81.4)	125 (77.2)	98 (77.2)	728 (81.5)
One parent alive	47 (12.2)	34 (15.5)	29 (17.9)	24 (18.9)	134 (15.0)
Orphan	11 (2.9)	7 (3.2)	8 (4.9)	5 (3.9)	31 (3.5)
*Parents’ marital status*					
Married/cohabiting	315 (86.5)	179 (87.3)	128 (85.9)	112 (88.9)	734 (87.0)110
Separated/widowed	49 (13.5)	26 (12.7)	21 (14.1)	14 (11.1)	(13.0)
*Guardian/caretaker*					
Parents	339 (92.1)	195 (91.6)	139 (90.8)	113 (90.4)	786 (91.5)
Others	29 (7.9)	18 (8.4)	14 (9.2)	12 (9.6)	73 (8.5)

### Prevalence of psychological distress

Out of the 906 students, 390 students had scores less than 20 on the K10 (psychological Distress Scale) and were therefore classified as normal (43%). The prevalence of mild psychological distress was (24.6%), moderate psychological distress was (18%) and severe psychological distress was (14.4%). Therefore, the overall prevalence of any psychological distress among study participants was 57%.

### Factors associated with psychological distress

The unadjusted logistic regression showed that age, religion, family background, type of school (single/mixed sex), mobile phone ownership, chronic illness, risky sexual behavior and substance use were significantly associated with psychological distress (Table 2).

**Table 2:** Factors associated with psychological distress

Variable	Unadjusted OR (95% CI)	*p*-value	Adjusted OR (95% CI)	*p*-value
*Age*				
Below 18 years	(ref)	0.007	(ref)	0.577
18 years and above	1.48 (1.11–1.97)		1.10 (0.78–1.55)	
*Gender*				
Female	(ref)	0.634	–	–
Male	1.07 (0.82–1.39)			
*Type of student*				
Boarding	1.25 (0.92–1.71)	0.150	–	–
Day	(ref)			
*Type of school*				
Private	1.06 (0.82–1.38)	0.649	–	–
Public	(ref)		1.94	
Mixed (both sex)	2.04 (1.38–3.01)	< 0.001	(1.19–3.15)	0.007**
Single-sex	(ref)		(ref)	
*Residence*				
Urban/town	(ref)	0.074	–	–
Sub-urban	1.29 (0.98–1.70)			
*Religion*				
Catholic	1.60 (1.04–2.46)		1.35 (0.81–2.26)	0.250
Moslem	1.15 (0.65–2.02)	0.042	1.01 (0.52–1.96)	0.986
Protestant	1.09 (0.68–1.75)		0.91 (0.52–1.50)	0.737
Pentecostal	1.18 (0.74–1.88)		1.09 (0.63–1.89)	0.760
Other	(ref)		(ref)	
*Family type*				
Both parent alive	(ref)	0.028	(ref)	
One parent alive	1.50 (1.02–2.20)		1.36 (0.89–2.09)	0.155
Orphan	1.47 (0.70–3.12)		1.28 (0.54–3.07)	0.575
*Guardian/caretaker*				
Parents	(ref)	0.574	–	–
Others	1.15 (0.71–1.88)			
*Parents’ marital status*				
Married/cohabiting	1.07 (0.71–1.60)	0.748	–	–
Separated/widowed	(ref)			
*Mobile phone ownership*				
No	(ref)	< 0.001	(ref)	0.062
Yes	1.73 (1.32–2.26)		1.35 (0.98–1.84)	
*Social support*				
No	(ref)	0.670	–	–
Yes	1.08 (0.76–1.53)			
*Exam failure*				
No	(ref)	0.136	–	–
Yes	1.28 (0.93–1.76)			
*Chronic illness*				
No	(ref)	< 0.001	(ref)	0.004**
Yes	1.89 (1.37–2.61)		1.68 (1.18–2.38)	
*Risky sex behaviour*				
No	(ref)	< 0.001	(ref)	0.042*
Yes	1.97 (1.38–2.83)		1.52 (1.02–2.28)	
*Substance use*				
No	(ref)	< 0.001	(ref)	< 0.001***
Yes	2.43 (1.82–3.24)		2.06 (1.49–2.84)	

Ref = reference

*p < 0.05; **p < 0.01; ***p < 0.001

In the adjusted logistic regression, students in mixed schools (AOR = 1.94; 95% CI 1.19–3.15), those who had reported having a chronic illness (AOR = 1.68; 95% CI 1.18–2.38), and students that engaged in risky sexual behavior (AOR = 1.52; 95% CI 1.02–2.28), or used psychoactive substances (AOR = 2.06; 95% CI 1.49–2.84) were significantly more likely to have psychological distress (Table 2).

## Discussion

The aim of the present study was to determine the prevalence of psychological distress and to identify the high-risk behaviors associated with psychological distress among secondary school adolescents in Mbarara Municipality, Uganda.

The overall prevalence of psychological distress was 57% with 24.6% mild psychological distress, 18% moderate psychological distress, and 14.4% severe psychological distress. Generally, previous studies have shown a high prevalence of mental health issues among adolescents in Uganda. The prevalence of psychological distress in the present study is consistent with the prevalence rates reported by some studies in Uganda. A recent study conducted among youths in Kampala, Uganda showed that the prevalence of psychological distress ranged from 51.3 to 91.2% although the study participants comprised orphans and youths experiencing sexual exploitation [13]. Another study conducted among adolescents living with HIV/AIDS in Uganda reported a comparable rate of 51% psychological distress [30]. Contrary to the present study findings, a study conducted among refugee adolescents in West Nile, Uganda reported a much lower prevalence of psychological distress (12.3%) [7]. The stress and pressure associated with secondary school education and high expectations from the teachers and parents or guardian of these students might explain the high prevalence of psychological distress among the school-going adolescents reported in the present study [42]. In addition to academic stress, undesirable life events seen in low-income countries could have contributed to the high prevalence of psychological distress in the present study population [27]. In comparison to studies from other countries, the present study’s finding is similar to the rates reported in Tanzania [12], Ethiopia [20], Egypt [43], India [44], Saudi Arabia [45], Canada [9] and China [46], but different from rates in Zambia [11], Morocco [47] and the USA [48]. The differences in the prevalence rates of psychological distress could be due to the diverse environment, culture and economic factors as well as varied study samples, assessment tools and methodology used in these studies.

In the present study, students who used psychoactive substances were more likely to report psychological distress compared to students who did not. This is consistent with a prior multi-country study where alcohol consumption was associated with psychological distress in school-going adolescents [18]. It can be hypothesized that the association between psychological distress and substance use among adolescents is attributed to the use of these substances as a self-medicating drug to help cope with stressors and/or life challenges. Furthermore, this may be due to the fact that adolescents are curious and are more likely to engage in risky behaviors such as alcohol consumption and smoking. Also, coupled with easy accessibility and availability of these substances as well as weak regulatory policies and enforcements in Uganda, school-going adolescents are exposed to substance use in early age and may be psychologically distressed due to the negative consequences of substance use [10, 11, 18–21]. Previous studies conducted in Tanzania [12], Ethiopia [20], Zambia [11], India [8], Norway [21] and Finland [49] have all shown similar results although a study in Canada reported that alcohol and cannabis use were not associated with psychological distress among adolescents [9].

The present study also found that adolescents who engaged in risky sexual activities were more likely to report psychological distress. This relationship is supported by previous studies conducted among orphaned youths and adolescents reporting sexual exploitation in Kampala, Uganda [13], school-going adolescents in Tanzania [12], adolescents in Sub-Saharan Africa [22], and adolescents in the US [17, 23, 24]. One possible explanation for this relationship could be that adolescents become distressed due to engaging in risky sexual behavior and they may be anxious about getting pregnant or impregnating someone, and having infectious diseases including HIV/AIDS, as well as difficulties in romantic relationships [24, 50, 51]. Another possible reason for this association could be that psychological distress may make it difficult for school-going adolescents to practice safer sex [52].

Students with the history of chronic health conditions were more likely to experience psychological distress compared to those who did not. This is in line with a research conducted among Ugandan adolescents living with HIV [53] and supported by other studies that examined the relationship between psychological distress and chronic health conditions [10, 54]. This could (among other factors) be attributed to adverse social circumstances and financial strain, reduced ability to cope with routine daily activities, and aggravated physical consequences such as pain and discomfort [55]. Apart from the high-risk behaviors and chronic health conditions identified in the present study, being a student in a mixed school (schools with both genders) was associated with psychological distress. Contrary to existing studies [8, 9, 20, 47, 56], age, gender, social support and academic performance were not associated with psychological distress in the present study. The samples and the scales used were likely to account for the differences.

### Limitations

The present study has limitations that should be considered when interpreting the results. The cross-sectional design of the present study does not allow the determination of any cause-effect relationships between the study variables. Also, a self-reported instrument was used to assess psychological distress with no diagnostic interview conducted to establish the presence of actual psychological distress, therefore, there may be reporting bias. Moreover, other self-report biases may have occurred including social desirability responses, and some questions require recall of past history which is prone to recall bias.

## Conclusions

A high prevalence of psychological distress was identified among secondary school students in Uganda in the present study. The finding indicates that over half of school-going adolescents have psychological distress as assessed using the K10. Moreover, the present study showed that attending mixed-sex schools, chronic health conditions, risky sexual behavior and substance use were associated with psychological distress. These findings suggest the need for policy makers and stakeholders in education and health sectors to institute mental health programs to prevent, control, and treat psychological distress among school-going adolescents. In addition, comprehensive substance use control policies such as laws against the sale of alcohol, cigarette, tobacco and other psychoactive substances to adolescents should be enacted and implemented. Also, integration of sexual, substance use and chronic health care services with mental health programs could facilitate the prevention of psychological distress among adolescents.

## Data Availability

The datasets used and/or analyzed during the present study are available from the corresponding author on reasonable request.
